# Clinico-pathological study of esophageal mucoepidermoid carcinoma: a 10-year survival from a single center

**DOI:** 10.1186/s12876-024-03215-w

**Published:** 2024-05-08

**Authors:** Yi Wang, Yajing Wu, Chen Zheng, Qihui Li, Wenpeng Jiao, Jianing Wang, Linlin Xiao, Qingsong Pang, Wencheng Zhang, Jun Wang

**Affiliations:** 1 Department of Radiation Oncology, the Fourth Hospital of Hebei Medical University, Shijiazhuang, 050011 China; 2 Department of Radiation Oncology, Tianjin Medical University Cancer Institute and Hospital, Tianjin, China

**Keywords:** Carcinoma, Mucoepidermoid, Diagnosis, Metastasis, Prognostic

## Abstract

**Background:**

Mucoepidermoid Carcinoma of the Esophagus (MECE) is a relatively rare tumor type, with most of the current data derived from case reports or small sample studies. This retrospective study reports on the 10-year survival data and detailed clinicopathological characteristics of 48 patients with esophageal MEC.

**Methods:**

Data were collected from 48 patients who underwent curative surgery for esophageal MEC at the Fourth Hospital of Hebei Medical University between January 1, 2004, and December 31, 2020. These were compared with contemporaneous cases of Esophageal Squamous Cell Carcinoma (ESCC) and Esophageal Adenocarcinoma (EAC). Using the Kaplan-Meier method and multivariate Cox regression analysis, we investigated the clinicopathological factors affecting the survival of patients with MEC.

**Results:**

The incidence of MECE was predominantly higher in males, with a male-to-female ratio of approximately 7:1. The mid-thoracic segment emerged as the most common site of occurrence. A mere 6.3% of cases were correctly diagnosed preoperatively. The lymph node metastasis rate stood at 35.4%. The overall 1-year, 3-year, 5-year, and 10-year survival rates for all patients were 85.4%, 52.1%, 37.0%, and 31.0%, respectively. Post 1:1 propensity score matching, no significant statistical difference was observed in the Overall Survival (OS) between MEC patients and those with Esophageal Squamous Cell Carcinoma (ESCC) and Esophageal Adenocarcinoma (EAC) (*P* = 0.119, *P* = 0.669). Univariate analysis indicated that T staging and N staging were the primary factors influencing the prognosis of esophageal MEC.

**Conclusions:**

MECE occurs more frequently in males than females, with the mid-thoracic segment being the most common site of occurrence. The rate of accurate preoperative endoscopic diagnosis is low. The characteristic of having a short lesion length yet exhibiting significant extramural invasion may be a crucial clinicopathological feature of MECE. The OS of patients with MEC does not appear to significantly differ from those with esophageal squamous carcinoma and adenocarcinoma.

## Introduction

Mucoepidermoid carcinoma (MEC) is a malignant tumor commonly found in the salivary glands, lacrimal glands, and bronchi, but its occurrence in the esophagus is extremely rare [1], representing only 0.05–2.2% of esophageal cancer cases [1–4]. The origin of esophageal MEC is still a matter of debate. It is histologically composed of a mixture of squamous, mucous, and intermediate cells. The rate of misdiagnosis under endoscopy is extremely high, with some reports even indicating a 0% accuracy rate in diagnoses [3, 5], making its identification even more challenging.

Owing to the infrequency of MECE occurrences, comprehensive research on its biological characteristics, therapeutic approaches, and prognostic outcomes is limited, particularly regarding long-term survival data. This study endeavors to meticulously evaluate the clinicopathological attributes and biological behavior of MECE, juxtaposing it with contemporaneous cases of ESCC and EAC. Survival disparities are examined post-propensity score matching. This research includes a cohort of 48 patients who underwent definitive surgical intervention for MECE at the Fourth Hospital of Hebei Medical University, representing one of the more substantial single-center datasets in this domain. Significantly, it provides the inaugural disclosure of a decade-long survival analysis for MECE patients.

## Patients and Methods

### Data resources and study population

From January 1, 2004, to December 31, 2020, 48 patients underwent curative surgical resection for MECE at the Fourth Hospital of Hebei Medical University. These cases constituted 0.38% (48/12,648) of all primary esophageal cancer surgeries performed at the institution during the same period. The male-to-female ratio was 7:1, with ages ranging from 45 to 78 years and a median age of 63 years. Based on the 8th edition of the TNM staging system for esophageal cancer published by the Union for International Cancer Control (UICC) in 2017, patients diagnosed prior to 2017 were restaged accordingly. Detailed baseline characteristics of the patients are presented in Table 1.

### Definition of lymph node metastasis

Lymph node metastasis rate (%) = number of cases with lymph node metastases confirmed by pathology / total number of cases × 100%.

### Therapeutic modalities

All the 48 patients underwent radical resection. Forty-two patients received Sweet operations, two received Ivor-Lewis operations, and four received MIE (Minimally Invasive Esophagectomy) operations. Among the 48 patients, 26 cases were treated with operation alone and 22 cases completed combined modality therapy, including 3 cases treated with neoadjuvant chemotherapy and 17 cases with adjuvant chemotherapy. Chemotherapy was commonly conducted for 1 to 4 cycles (median: 2 cycles). Postoperative adjuvant radiotherapy was performed in 3 cases, and the dose fraction was 45 Gy, 50 Gy and 54 Gy with 2 Gy per fraction, 5 fractions a week. (Table 2)

### Follow-up

The follow-up for the entire cohort of patients was concluded on October 15, 2023, with a median follow-up period of 52 months. Two cases were lost to follow-up, resulting in a follow-up rate of 95.8%. Overall survival time was defined as the duration from the date of surgery to the date of death or the last follow-up.

### Statistical method

Data analysis was conducted using the Statistical Product and Service Solutions (SPSS) software, version 27.0. Categorical variables were compared using the Pearson Chi-square test. Overall survival (OS) was calculated using the Kaplan-Meier method and assessed with the Log-rank test. Multivariate regression analysis was employed to identify independent prognostic factors using the Cox proportional hazards model. Propensity score matching (PSM) was executed using a 1:1 nearest neighbor matching method. Covariates included gender, age, tumor location, tumor length, T staging, N staging, and comprehensive treatment. A *p*-value of less than 0.05 was considered statistically significant.

## Results

### Preoperative endoscopic diagnosis

Preoperative endoscopic diagnosis of MECE was confirmed in only 3 cases, resulting in a diagnostic accuracy of merely 6.3% (3/48). Among the misdiagnosed cases, 28 were incorrectly identified as squamous cell carcinoma, 8 as adenocarcinoma, 4 as poorly differentiated carcinoma, 3 as adenosquamous carcinoma, and 2 as mucinous gland carcinoma.

### Immunohistochemistry

Immunohistochemical examinations were performed in 19 of the all 48 patients, including 3 cases with PSA positive, 9 cases with p63 positive, 10 cases with CEA positive, 10 cases with CK positive, 10 cases with CK5/6 positive and 5 cases with CK7 positive.

### Lymph node metastasis

Among the 48 patients, 17 experienced lymph node metastasis, resulting in a lymph node metastasis rate of 35.4% (17/48). Further analysis of the lymph node metastasis rate, 0% for T1 stage (0/3), 25% for T2 stage (1/4), 25% for T3 stage (8/32), and 88.9% for T4 stage (8/9), with a statistical significance(*P* = 0.002). For lesions length <5 cm, the lymph node metastasis rate was 14.7% (5/34), compared to 85.7% (12/14) for lesions ≥ 5 cm, with a highly significant difference (*P* < 0.001).

### Recurrence and Metastasis

Up to the last follow-up date, there were 17 cases presenting with local / regional recurrences and 13 cases with distant metastases (including 6 cases with lung metastases, 4 cases with liver metastases, 2 cases with abdominal lymph node metastases and 1 case with bone metastasis). One patient presented supraclavicular lymph node metastasis in 18 months after operation, but survived more than 12 years after local radiotherapy.

### Survival Analysis

As of the follow-up date, 32 patients died, including 27 cases of recurrence / metastasis and 5 cases of non-tumor factors (2 cases of malnutrition, 1 case of respiratory failure, 1 case of multiple organs failure, and 1 case of unknown cause). The median survival time of all patients was 48 months (6.5–184 months), and the 1-, 3-, 5-and 10-year survival rates were 87.5%, 54.2%, 39.2% and 33.6%, respectively.

**Table 1:** Baseline characteristics of patients

Characteristics	*n*(%)	Charactersitics	*n*(%)
Gender		pT stage	
Male	42(87.5%)	T_1_	3(6.3%)
Female	6(12.5%)	T_2_	4(8.3%)
Age		T_3_	32(66.7%)
<60y	15(31.3%)	T4	9(18.7%)
≥ 60y	33(68.7%)	pN stage	
Lesion location		N0	31(64.6%)
Upper thoracic	3(6.3%)	N+	17(35.4%)
Middle thoracic	34(70.8%)	pTNM stage	
Lower thoracic	11(22.9%)	I	6(12.5%)
Lesion length		II	25(52.1%)
<5 cm	34(70.8%)	III	11(22.9%)
≥ 5 cm	14(29.2%)	IVa	6(12.5%)
Vascular invasion		Nerve invasion	
No	43(89.6%)	No	41(85.4%)
Yes	5(10.4%)	Yes	7(14.6%)

#### Comparison of Clinicopathological Characteristics and Survival between MECE, ESCC, and EAC Patients

From January 1, 2004, to December 31, 2020, among the 12,648 patients who underwent curative surgical resection at our hospital, 93.83% (11,868/12,648) were pathologically diagnosed with Esophageal Squamous Cell Carcinoma (ESCC), and 2.71% (343/12,648) with Esophageal Adenocarcinoma (EAC). Considering the large number of ESCC patients, we employed a random sampling method, selecting 20% (2,530 patients) for analysis. Patients with incomplete data or lacking follow-up information were excluded, resulting in the inclusion of 2,348 ESCC patients and 307 EAC patients for the final analysis.

**Table 2:** Patients treatments

Treatment	Mode	Number(%)	Details	Number(%)
Operation	Sweet	42(87.5%)	Supra-arch anastomosis	34(81.0%)
			Subarch anastomosis	6(14.2%)
			Cervical anastomosis	2(4.8%)
	Ivor-Lewis	2(4.2%)	Supra-arch anastomosis	2(100%)
	MIE	4(8.3%)	Supra-arch anastomosis	1(25.0%)
			Cervical anastomosis	3(75.0%)
Treatment	operation alone	26(54.2%)	—	—
	combined therapy	22(45.8%)	Neoadjuvant chemotherapy	3(13.6%)
			Adjuvant chemotherapy	16(72.7%)
			Adjuvant radiochemotherapy	1(4.5%)
			Adjuvant radiotherapy	2(9.1%)
Chemotherapy	Adjuvant chemotherapy	20(41.7%)	FP	12(60.0%)
			FOLFOX	2(10.0%)
			TP	6(30.0%)

**Fig. 1:**
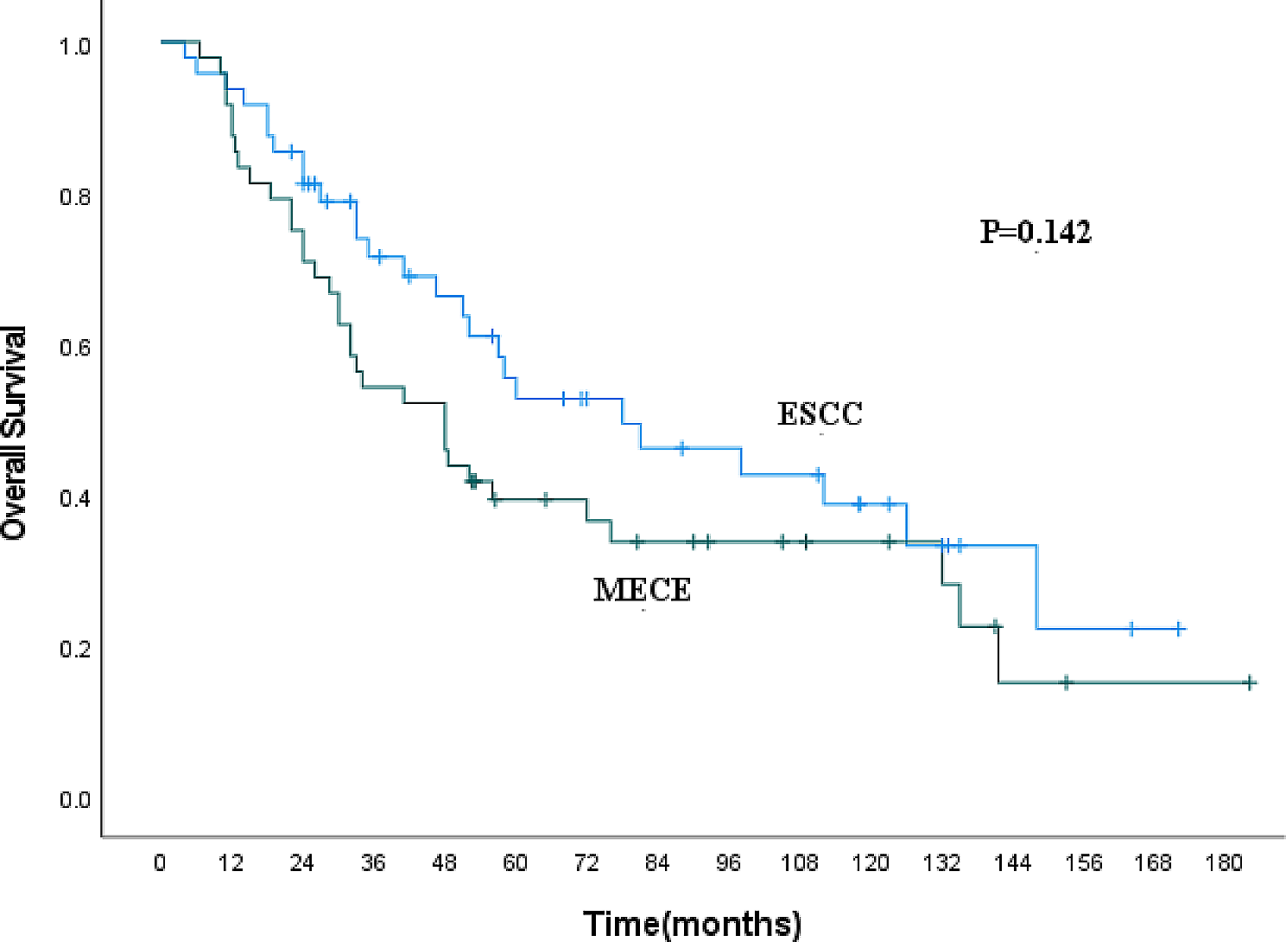
Kaplan-Meier curve for overall survival of MECE and ESCC patients

The clinicopathological characteristics of MECE and ESCC patients are presented in Table 3, showing differences in gender, lesion length, and pT staging between the two groups. Compared to ESCC, MECE was more prevalent in male patients (87.5% vs. 74.3%, *P* = 0.038). MECE patients had shorter lesion lengths (< 5 cm) more frequently (70.8% vs. 44.5%, *P* < 0.001), but a lower proportion of early T stages (T1 + T2) (14.6% vs. 31.8%, *P* = 0.011). To control for the potential influence of baseline characteristics on survival analysis, we performed a 1:1 propensity score matching for MECE and ESCC patients, considering characteristics like gender, age, lesion location, lesion length, pT stage, pN stage, and comprehensive treatment. After matching, there were no significant differences in the clinicopathological characteristics between MECE and ESCC patients (Table 3). The 1-year, 3-year, 5-year, and 10-year OS for ESCC patients were 93.7%, 71.4%, 52.6%, and 38.6%, respectively, showing no statistical difference with MECE patients (*P* = 0.142, Fig. 1).

**Table 3:** Clinicopathological characteristics of MECE and ESCC patients in the original and matched cohorts

Characteristics	Original cohor		*P* value	Matched cohort		*P* value
	MECE (*n* = 48)	ESCC (*n* = 2348)		MECE (*n* = 48)	ESCC (*n* = 48)	
Gender						
Male	42(87.5%)	1745(74.3%)	0.038	42(87.5%)	42(87.5%)	1.000
Female	6(12.5%)	603(25.7%)		6(12.5%)	6(12.5%)	
Age						
<60y	15(31.3%)	958(40.8%)	0.184	15(31.3%)	14(29.2%)	0.824
≥ 60y	33(68.7%)	1392(59.3%)		33(68.7%)	34(70.8%)	
Lesion location						
Upper thoracic	3(6.3%)	228(9.7%)	0.491	3(6.3%)	2(4.2%)	0.885
Middle thoracic	34(70.8%)	1712(72.9%)		34(70.8%)	34(70.8%)	
Lower thoracic	11(22.9%)	409(17.4%)		11(22.9%)	12(25.0%)	
Lesion length						
<5 cm	34(70.8%)	1045(44.5%)	<0.01	34(70.8%)	34(70.8%)	1.000
≥ 5 cm	14(29.2%)	1303(55.5%)		14(29.2%)	14(29.2%)	
pT stage						
T_1_-T2	7(14.6%)	747(31.8%)	0.011	7(14.6%)	7(14.6%)	1.000
T3-T4	41(85.4%)	1601(68.2%)		41(85.4%)	41(85.4%)	
pN stage						
N0	31(64.6%)	1280(54.5%)	0.165	31(64.6%)	31(64.6%)	1.000
N+	17(35.4%)	1068(45.5%)		17(35.4%)	17(35.4%)	
combined therapy						
Yes	22(45.8%)	1334(56.8%)	0.129	22(45.8%)	21(43.8%)	0.837
No	26(54.2%)	1014(43.2%)		26(54.2%)	27(56.3%)	

**Fig. 2:**
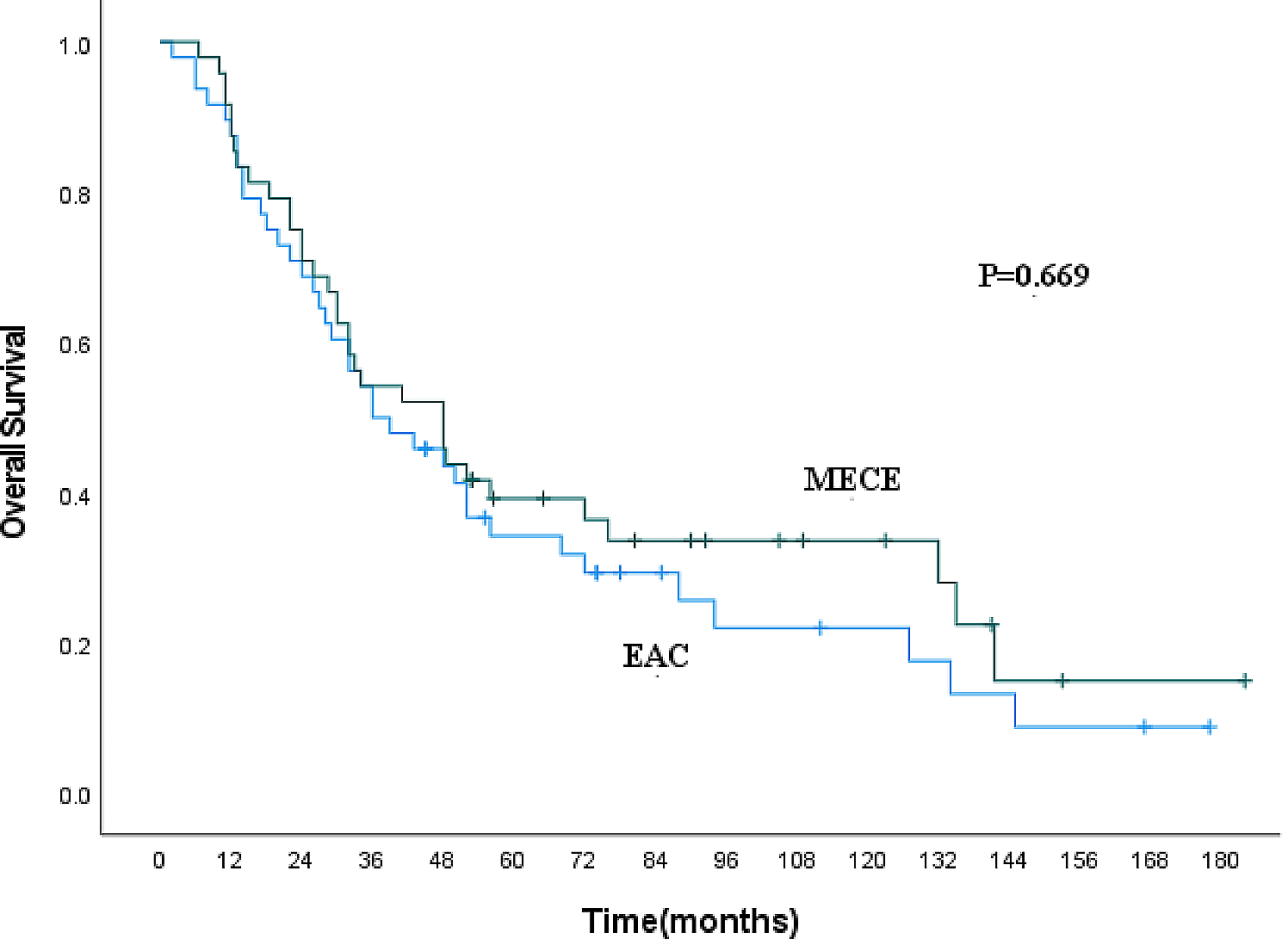
Kaplan-Meier curve for overall survival of MECE and EAC patients

Regarding MECE and EAC patients, the clinicopathological features are outlined in Table 4, revealing differences in lesion site, lesion length, pT stage, and pN stage between the groups. MECE had a higher incidence in the mid-esophageal segment compared to EAC (70.8% vs. 50.7%), while EAC was more commonly found in the lower esophagus (22.9% vs. 43.8%, *P* = 0.023). A larger proportion of MECE patients had lesions less than 5 cm (70.8% vs. 46.8%, *P* = 0.002). Fewer MECE patients were in the early T stages (T1 + T2) (14.6% vs. 32.8%, *P* = 0.010), and more had lymph node metastasis (35.4% vs. 21.4%, *P* = 0.034). After 1:1 propensity score matching, there were no significant differences in the clinicopathological features between MECE and EAC patients (Table 4). The 1-year, 3-year, 5-year, and 10-year OS rates for EAC patients were 87.5%, 54.2%, 34.2%, and 22.0%, respectively, without any statistical difference (*P* = 0.669, Fig. 2).

**Table 4:** Clinicopathological characteristics of EAC and ESCC patients in the original and matched cohorts

Characteristics	Original cohor		*P* value	Matched cohort		*P* value
	MECE (*n* = 48)	EAC (*n* = 307)		MECE (*n* = 48)	EAC (*n* = 48)	
Gender						
Male	42(87.5%)	249(81.1%)	0.625	42(87.5%)	42(87.5%)	1.000
Female	6(12.5%)	58(18.9%)		6(12.5%)	6(12.5%)	
Age						
<60y	15(31.3%)	127(41.3%)	0.183	15(31.3%)	14(29.2%)	0.824
≥ 60y	33(68.7%)	180(58.7%)		33(68.7%)	34(70.8%)	
Lesion location						
Upper thoracic	3(6.3%)	17(5.5%)	0.023	3(6.3%)	3(6.3%)	1.000
Middle thoracic	34(70.8%)	156(50.7%)		34(70.8%)	34(70.8%)	
Lower thoracic	11(22.9%)	134(43.8%)		11(22.9%)	11(22.9%)	
Lesion length						
<5 cm	34(70.8%)	144(46.8%)	0.002	34(70.8%)	34(70.8%)	1.000
≥ 5 cm	14(29.2%)	163(53.2%)		14(29.2%)	14(29.2%)	
pT stage						
T_1_-T2	7(14.6%)	101(32.8%)	0.010	7(14.6%)	7(14.6%)	1.000
T3-T4	41(85.4%)	206(67.2%)		41(85.4%)	41(85.4%)	
pN stage						
N0	31(64.6%)	241(78.6%)	0.034	31(64.6%)	33(68.8%)	0.665
N+	17(35.4%)	66(21.4%)		17(35.4%)	15(31.3%)	
combined therapy						
Yes	22(45.8%)	124(40.3%)	0.476	22(45.8%)	21(43.8%)	0.837
No	26(54.2%)	183(59.7%)		26(54.2%)	27(56.3%)	

#### Univariate analysis of MECE survival

**Fig. 3:**
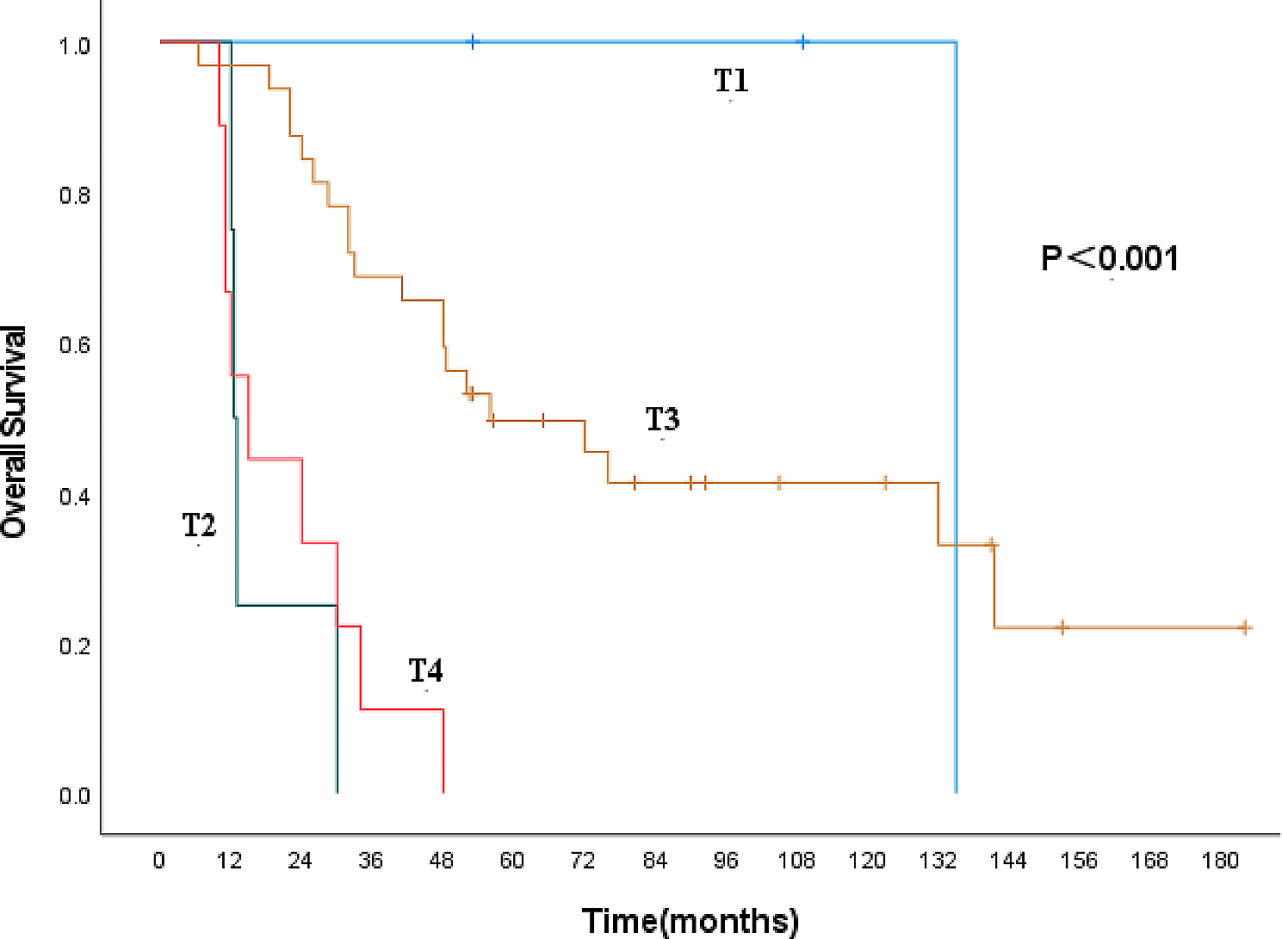
Kaplan-Meier curves for overall survival according to pT category

**Fig. 4:**
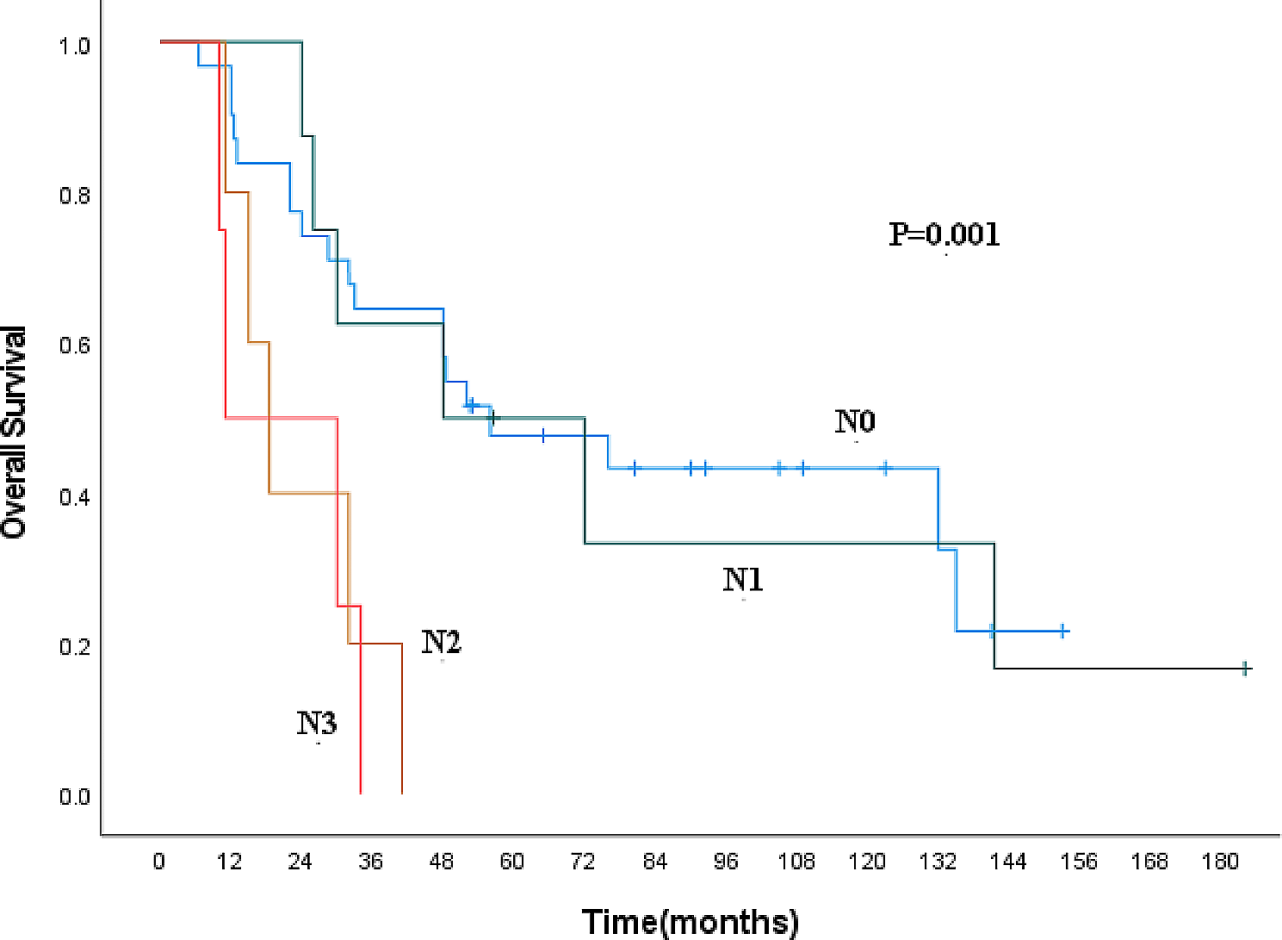
Kaplan-Meier curves for overall survival according to pN category

Nine clinicopathological factors, including gender, age, lesion location, lesion length, pT stage, pN stage, presence of vascular invasion, nerve invasion, and whether combined therapy was administered, were included in the univariate analysis (Table 5). pT stage and pN stage were identified as influencing factors (Figs. 3 and 4). Age, gender, pT stage, and pN stage were then incorporated into the multivariate analysis, which revealed that gender and pN stage are independent prognostic factors for patients with MECE (Table 6).

**Table 5:** Univariate analysis of various potential prognostic factors associated with OS

Factor	n(%)	Overall Survival rate (%)				χ^2^	*P*
		1-y	3-y	5-y	10-y		
Gender							
Male	42(87.5)	90.5	57.1	42.6	6.5	2.162	0.141
Female	6(12.5)	66.7	33.3	16.7	16.7		
Age							
<60y	33(68.8)	90.9	57.6	45.5	40.9	1.476	0.224
≥ 60y	15(31.2)	80.0	46.7	26.7	20.0		
Lesion location							
Upper thoracic	3(6.3)	100.0	66.7	33.3	33.3	1.542	0.463
Middle thoracic	34(70.8)	82.4	50.0	35.3	28.9		
Lower thoracic	11(22.9)	100	63.6	54.5	54.5		
Lesion length							
<5 cm	14(29.2)	78.6	42.9	28.6	21.4	2.021	0.155
≥ 5 cm	34(70.8)	91.2	58.8	43.4	39.1		
pT stage							
T1	3(6.3)	100.0	100.0	100.0	100.0	31.573	<0.001
T2	4(8.3)	75.0	0	0	0		
T3	32(66.6)	96.9	68.8	49.6	41.3		
T4	9(18.8)	55.6	11.1	0	0		
pN stage							
N0	31(64.6)	90.3	64.5	47.6	43.3		
N1	8(16.7)	100	62.5	50.0	33.3	8.416	0.015
N2	5(10.4)	80.0	20.0	0	0		
N3	4(8.3)	50.0	0	0	0		
Vascular invasion							
No	43(89.6)	86.0	53.5	41.4	35.1	0.105	0.746
Yes	5(10.4)	100.0	60.0	20.0	20.0		
Nerve invasion							
No	41(85.4)	85.4	53.7	36.4	31.2	0.773	0.379
Yes	7(14.6)	100	57.1	57.1	57.1		
combined therapy							
Yes	22(40.7)	81.8	50.0	40.9	29.2	0.250	0.617
No	26(52.3)	92.3	57.7	37.6	37.6		

**Table 6:** Multivariate analysis of survival-related factors in MECE patients

Factor	B	SE	Wald	*P*-value	Exp(B)	95.0% CI
Gender	1.073	0.530	4.106	0.043	2.925	1.036–8.262
Age	0.300	0.374	0.641	0.424	1.349	0.648–2.811
pT stage	0.381	0.341	1.249	0.264	1.463	0.751–2.853
pN stage	0.541	0.204	7.003	0.008	1.717	1.150–2.562

## Discussion

MEC was the most common malignant tumor in salivary glands [6], but it was rather rare in esophagus. MECE was a rare subtype of esophageal cancer, and is mostly reported in Asian population [7]. From the data from our center, we noticed that MECE accounted for 0.38% (48/12,648) of patients with primary esophageal cancer.

The existing literature on MECE indicates a median age of onset around 60 years and a predilection for the mid to lower segments of the esophagus, characteristics seemingly similar to those of esophageal squamous carcinoma. In our study, the median age of the patients was 63 years (ranging from 45 to 78 years), with 93.8% (45/48) of the tumors located in the mid to lower thoracic segments. Our hospital’s data shows a male-to-female ratio of 7:1 (42/6) for MECE, higher than the 3:1 ratio (1745/603) observed in ESCC patients in the same period. This aligns with the findings of Fegelman [8], who reported on 20 cases of MECE with an average age of 65.7 years, 80% occurring in the mid to lower segments, and a male-to-female ratio of 9:1. In a retrospective analysis of 87 MECE cases from various studies. Turkyilmaz [1] noted an average age of onset at 61.8 years, with 89.7% (78/87) of cases in the mid to lower thoracic esophagus, and a male-to-female incidence ratio of 3.8:1 (69:18). Similarly, Chen [3] reported on 36 cases of MECE with a median age of 58 years (ranging from 40 to 78 years), 86.1% in the mid to lower segments, and a male-to-female ratio of 3:1 (27:9).

The origin of MECE has been a topic of debate among scholars. Most believe that MECE originates from the ductal epithelium and acinar cells of the submucosal glands. Previously, the World Health Organization (WHO) classified MEC as a subtype of esophageal adenocarcinoma [9]. However, some scholars argue that MECE might originate from squamous epithelium, considering its extension into and infiltration of squamous tissue, predilection for certain sites, close association with dysplastic epithelial areas, and prognosis similar to squamous cell carcinoma [10]. As reported by Ozawa [5] and Chen [3], who noted a 0% accuracy rate for preoperative endoscopic diagnosis of MECE, Wang [11] observed that in a study of 47 patients, only one was diagnosed with MECE preoperatively via endoscopy, while the others were predominantly misdiagnosed as squamous cell carcinoma. In our series, the preoperative endoscopic diagnosis accuracy was only 6.3% (3/48), with MECE more frequently misdiagnosed as squamous carcinoma than adenocarcinoma (28 cases vs. 8 cases). Notably, since 2019, WHO has classified MECE as a distinct category. Given the complexity of MEC tumors and the limitations of preoperative biopsy, enhancing the accuracy of preoperative diagnosis through multiple endoscopic biopsies and the incorporation of immunohistochemical techniques is crucial.

Although no specific immunohistochemical markers can entirely differentiate MECE from other types of esophageal cancer, the expression of p63, CEA, and CK can aid in improving the diagnosis and differential diagnosis of MECE. In the study by Hagiwara [2], the positive expression rate of CEA in MEC was significantly higher than in SCC (100% vs. 49%, *P* < 0.05). Studies by Huo Z [12] and Zhang [13] indicated that pulmonary mucoepidermoid carcinomas show high positive expression rates of immunohistochemical markers CK7, p63, and CK5/6, CK. In our study, 19 cases underwent immunohistochemical examination, revealing that the positive expression rates of p63, CEA, and CK, CK5/6 were all around 50%. A single marker may not accurately reflect the complexity of the tumor, but the combined detection of multiple markers can effectively enhance the diagnostic value of tumor markers, which is particularly crucial in rare diseases like MECE.

In the comparison of clinicopathological characteristics between MECE patients and those with ESCC and EAC, it was found that 70.8% of MECE patients had lesions <5 cm, indicating shorter postoperative pathologic lesion lengths compared to squamous cell carcinoma and adenocarcinoma patients. The local invasion of the tumor was more severe in MECE, with T3 + T4 stages accounting for 85.4%, significantly higher than the 68.2% in ESCC and 67.2% in EAC during the same period (*P* = 0.011, *P* = 0.010). The characteristic of a short lesion length but extensive extramural invasion might be an important clinicopathological feature of MECE, which requires further verification in subsequent studies.

Lymph node metastasis is also a crucial indicator of tumor invasiveness. A literature review by Kumagai [14] of multiple studies from different hospitals and countries revealed a lymph node metastasis rate of 48.9% (45/92) in 92 MECE patients. Chen [3] and Wang [11] reported lymph node metastasis rates of 22.3% and 25.0%, respectively, significantly lower than the 49.4% in ESCC patients (*P* < 0.001). Our study showed a lymph node metastasis rate of 35.4% (17/48) in MECE patients, which did not significantly differ from that in ESCC (45.5%, *P* = 0.165) but was higher than in EAC (21.4%, *P* = 0.034). Further analysis indicated that the lymph node metastasis rate increased with higher T stages and longer lesion lengths (*P* < 0.05), demonstrating the impact of the primary tumor’s extent of extramural invasion and length on lymph node metastasis.

Some studies demonstrated that the prognosis of primary MECE was worse than that of traditional squamous cell carcinoma [2, 15, 16]. The 5-year survival rate was generally between 23.6% and 27.7% [3, 14, 17]. Most patients died of local recurrences or distant metastases of the tumors [17]. Hagiwara [2] reported 8 cases of MECE, of which 7 were with stage III diseases. The results showed that 4 cases died of distant metastases and 2 cases died of local recurrences within 2 years after operation. The median survival time was 10.8 months (4–24 months), which was significantly shorter than that of patients with squamous cell carcinoma (32.1 months) (*P* < 0.05). Chen [3] pointed out that the 5-year survival rate of MECE patients was lower than that of SCC patients (25.8%vs 39.2%). However, more recent studies have shown different results. Wang [11] and colleagues conducted a propensity score-matched analysis comparing surgically resected MECE with esophageal squamous carcinoma, revealing that the 5-year overall survival rate for MECE patients (58 cases) was 55.2%, similar to 61.9% in ESCC patients (*P* = 0.399). In our data, the 5-year OS for MECE was 39.2%, showing no statistical difference with matched ESCC patients (54.5%, *P* = 0.119), aligning with Wang et al.‘s findings. The median survival period was 38.0 months, significantly higher than reported by Hagiwara et al., likely due to the earlier staging of patients in our study, with stages I and II comprising 64.6%. Notably, in our study, MECE and EAC patients were also matched using propensity scores, and no statistical difference in survival rates was observed (*P* = 0.669). The survival of these two groups appeared to be more closely aligned, warranting further research. This study is the first to report a 10-year survival rate (31.0%) for MECE patients both domestically and internationally, indicating that the long-term survival prospects for MECE are reasonably favorable.

The value of adjuvant therapy after surgery for MECE was unclear. Turkyilmaz [1] analyzed 87 cases of esophageal mucoepidermoid carcinoma, including 62 cases undergoing operation alone, 6 cases treated with operation and adjuvant chemotherapy, 4 cases with operation and radiotherapy, 3 cases with operation and chemoradiotherapy and 12 cases with non-operative treatment. the median survival time was 13 months. The survival time of 3 patients receiving radiotherapy and chemotherapy were 54, 95 and 111 months respectively, suggesting that radiotherapy and chemotherapy might prolong the survival of patients. However, other reports have failed to confirm the benefits of radiotherapy and chemotherapy. For instance, Chen [3] analyzed the survival data of 36 patients with MECE, including 26 patients with operation alone and 10 patients with operation and postoperative radiotherapy. There was no significant difference in overall survival between the two groups (*P* > 0.05). Kumagai [14] pointed out that there was no significant difference in survival time between operation alone group (*n* = 65) and additional chemotherapy / radiotherapy group (*n* = 28). This study calculated the 1 -, 3-and 5-year overall survival rates of patients in the operation alone group were 88.5%, 53.8% and 34.3%, respectively, which showd no significant difference from those of patients in the adjuvant treatment group (81.8%, 50.0%, 40.0%). However, 1 case developed supraclavicular lymph node metastasis in 18 months after operation and survived more than 12 years after radiotherapy, indicating that timely salvage treatment after progression might play a positive role in improving the survival of patients. Due to the low incidence of MECE, all the current studies were retrospective with a small sample size. Multi-center clinical data were encouraged to summarized and analyzed to explore the optimal treatment modality in depth.

Due to the extremely low incidence of MECE, even though our study represents one of the larger single-center case series both domestically and internationally, there are limitations to consider. These include the long span of years covered by the cases, a lack of standardized surgical approaches, insufficient numbers of lymph nodes dissected, and a paucity in the number of pathological immunohistochemical analyses. Additionally, the small number of cases may lead to data bias, which warrants clinical attention. In the process of propensity score matching for ESCC cases, we were guided by the research of Fortin [18], which indicates that a subsample extracted from a large dataset is representative if it constitutes more than 10% of the total. Consequently, we randomly selected a 20% subsample for statistical analysis. Whether this subsample accurately reflects the entire dataset remains to be further verified and substantiated.

In summary, MECE is a rare disease that is often misdiagnosed preoperatively, with surgery being the primary mode of treatment. The lesions tend to be relatively short in length, with significant local tumor invasion. However, the long-term survival prospects are relatively good, and overall survival (OS) appears to be similar to that of squamous and adenocarcinomas, providing a reference for clinical practice. Nonetheless, the difficulty in conducting prospective studies on MECE presents significant challenges in exploring its biological behavior and identifying optimal treatment strategies. It is recommended to collaborate across multiple centers, expand the sample size, and integrate clinical data. Additionally, delving into the genomic/biological aspects of its origin and characteristics may further improve prognosis and understanding of this complex disease.

## Data Availability

All data generated or analyzed during this study are included in this article. Further enquiries can be directed to the corresponding author.
